# Comparative ribosome profiling reveals distinct translational landscapes of salt-sensitive and -tolerant rice

**DOI:** 10.1186/s12864-021-07922-6

**Published:** 2021-08-12

**Authors:** Xiaoyu Yang, Bo Song, Jie Cui, Lina Wang, Shuoshuo Wang, Linlin Luo, Lei Gao, Beixin Mo, Yu Yu, Lin Liu

**Affiliations:** 1 Guangdong Provincial Key Laboratory for Plant Epigenetics, Longhua Bioindustry and Innovation Research Institute, College of Life Sciences and Oceanography, Shenzhen University, Shenzhen, 518060 China; 2 Shenzhen Branch, Guangdong Laboratory for Lingnan Modern Agriculture, Genome Analysis Laboratory of the Ministry of Agriculture, Agricultural Genomics Institute at Shenzhen, Chinese Academy of Agricultural Sciences, Shenzhen, 518124 China; 3 College of Horticulture Science and Engineering, Shandong Agricultural University, Tai’an, 271018 China

**Keywords:** *O. sativa*, Ribosome profiling, Ribosome stalling, Salt stress, Translation

## Abstract

**Background:**

Soil salinization represents a serious threat to global rice production. Although significant research has been conducted to understand salt stress at the genomic, transcriptomic and proteomic levels, few studies have focused on the translatomic responses to this stress. Recent studies have suggested that transcriptional and translational responses to salt stress can often operate independently.

**Results:**

We sequenced RNA and ribosome-protected fragments (RPFs) from the salt-sensitive rice (*O. sativa* L.) cultivar ‘Nipponbare’ (NB) and the salt-tolerant cultivar ‘Sea Rice 86’ (SR86) under normal and salt stress conditions. A large discordance between salt-induced transcriptomic and translatomic alterations was found in both cultivars, with more translationally regulated genes being observed in SR86 in comparison to NB. A biased ribosome occupancy, wherein RPF depth gradually increased from the 5′ ends to the 3′ ends of coding regions, was revealed in NB and SR86. This pattern was strengthened by salt stress, particularly in SR86. On the contrary, the strength of ribosome stalling was accelerated in salt-stressed NB but decreased in SR86.

**Conclusions:**

This study revealed that translational reprogramming represents an important layer of salt stress responses in rice, and the salt-tolerant cultivar SR86 adopts a more flexible translationally adaptive strategy to cope with salt stress compared to the salt susceptible cultivar NB. The differences in translational dynamics between NB and SR86 may derive from their differing levels of ribosome stalling under salt stress.

**Supplementary Information:**

The online version contains supplementary material available at 10.1186/s12864-021-07922-6.

## Background

Soil salinization is one of the most serious environmental stresses facing modern agriculture, and it endangers over one-fifth of irrigated soil worldwide [1]. Crop yield is being greatly limited by soil salinization, and there is an urgent need to improve agricultural production to meet the continuously increasing global population [2]. Salinization is mainly caused by excessive accumulation of Na^+^ and Cl^−^ in soil and inhibits crop growth and development via Na^+^ toxicity and osmotic stress [3]. To survive under salt stress, plants have evolved a series of conserved acclimation mechanisms, such as the salt overly sensitive (SOS) pathway and reactive oxygen species (ROS) scavenging systems, for maintaining ion homeostasis and the integrity of cell membranes [3]. Several genes or quantitative trait loci (QTLs) contributing to salt tolerance have been identified in crop plants, including rice [4–6], maize [7–9] and tomato [10–12], but few genes have been successfully cloned. Therefore, a better mechanistic understanding of adaptive responses to salt stress is necessary for improving crop salt tolerance.

In the past few decades, the rapid development of high-throughput methodologies, such as next-generation sequencing and mass spectrometry, has made the analysis of genomes, transcriptomes and proteomes more feasible. Omics studies have unveiled transcriptional regulation networks under salt stress in crop plants such as rice [13–17]. However, gene expression is regulated not only at the transcriptional level but also at the translational level through microRNAs [18, 19], ribosome stalling [20, 21] and other mechanisms. In contrast to the large amount of research at the genomic, transcriptomic and proteomic levels, very few studies have assessed translatomics under salt stress condition. It is increasingly evident that transcriptional and translational responses to stresses are relatively independent processes [22–24], and dysregulation at the translational level can lead to a series of developmental abnormalities, disorders and diseases [25, 26]. Mass spectrometry provides the opportunity to identify translational products directly, but it is limited to an incomplete set of proteins, lacks information on translational dynamics and typically does not capture peptides that are shorter than 150 amino acids [27].

Ribosome profiling (ribo-seq) is a technology that can monitor in vivo RNA translation dynamics globally and quantitatively by high-throughput sequencing of ribosome-protected mRNA fragments (RPFs) [28]. To perform ribosome profiling, polysomes are digested by ribonucleases to obtain RPFs that are used for library construction. By sequencing the RPF library and the corresponding RNA library, a variety of information about in vivo translation can be obtained, such as the coverage of ribosomes on a transcript, the translation efficiency of genes, and the global profile of translated open reading frames (ORFs) under a given growth condition. Initial ribo-seq protocols in *Arabidopsis* revealed relatively weak 3-nt periodicity, a unique feature of translated regions distinct from free or untranslated mRNAs [22, 29, 30]. Recently, improved protocols that generate RPFs with strong 3-nt periodicity were developed in *Arabidopsis* [31] and tomato [32], paving the way for uncovering translational regulation mechanisms underlying plant growth, development and acclimation to environmental stresses.

Rice is one of the most important staple crops and is regularly consumed by over half of the world population. Rice is also a salt-sensitive crop, with seedling and reproductive stages being more susceptible to salt stress than other developmental stages [33]. Increasing soil salinization has severely threatened global rice production by reducing yield and lowering grain quality [34]. A significant amount of research has been conducted to better understand salt-tolerant rice cultivars, with the ultimate goal of engineering plants with improved salt tolerance. Thus far, over 70 QTLs, including several major ones such as *SKC1*, have been identified as salt tolerance-associated loci in rice [4, 35, 36]. Additionally, some genes that are involved in adaptive responses to salt stress have been characterized by analyzing salt tolerance-related mutants [2, 5, 6]. Moreover, a number of transcriptomic analyses have uncovered salt stress-related transcriptional networks [17, 37, 38]. However, since transcriptional and translational regulation of gene expression is largely independent, additional information is needed regarding translational changes during salt stress.

In this study, RNA-seq and ribo-seq were performed with seedling shoots of the salt-sensitive rice (*O. sativa* L.) cultivar ‘Nipponbare’ (NB) and the salt-tolerant cultivar ‘Sea Rice 86’ (SR86) under normal and salt stress conditions. Significant differences were found in the transcriptomic and translatomic responses to salt stress of each cultivar, suggesting that translational reprogramming represents a relatively independent layer of salt stress responses. NB and SR86 adopted different translational adaptive strategies under salt stress and a stronger translational response was observed in SR86. A biased ribosome occupancy along coding regions was observed and this phenomenon increased during salt stress, particularly in SR86, suggesting that more dynamic ribosomes were present in the salt-tolerant cultivar. Furthermore, the strength of ribosome stalling, a regulatory mechanism at the translation elongation phase, was accelerated in NB but decreased in SR86 under salt stress, providing a possible explanation for the divergent ribosome dynamics between NB and SR86. Our results shed new light on the translational acclimation of rice to salt stress and provide potential targets for genetic manipulation at the translational level to improve rice salt tolerance.

## Results

### Library construction with RPFs from NB and SR86

**Fig. 1:**
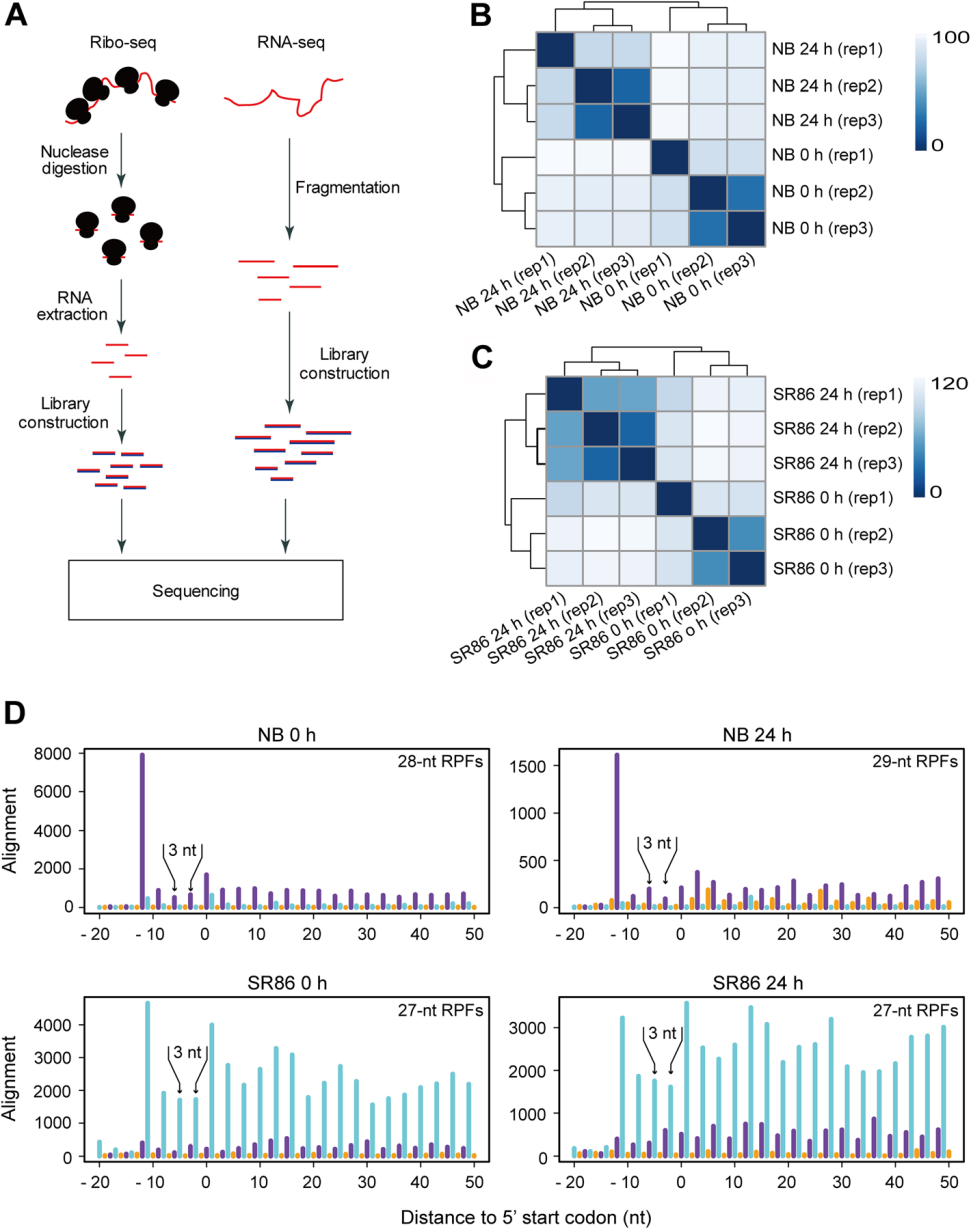
Library construction and sequencing for transcriptome and translatome profiling of ‘Nipponbare’ (NB) and ‘Sea Rice 86’ (SR86). **a** Schematic illustration for the construction of RNA-seq and ribo-seq libraries. **b** Clustering analysis of translatomic datasets from NB under normal (0 h) and salt stress (24 h) conditions. **c** Clustering analysis of translatomic datasets from SR86 under normal (0 h) and salt stress (24 h) conditions. The three biological repeats are represented by “rep 1”, “rep 2” and “rep 3”. The color schemes indicate Euclidean distances between samples measured by DESeq2-normalized read counts. **d** Metagene analysis of RPFs from NB and SR86 under normal (0 h) and salt stress (24 h) conditions. Lines at positions of frame 0 (the main frame based on the annotated start codon), 1 and 2 are colored in purple, cyan and orange, respectively

To determine the rice translational landscape, we performed ribo-seq and polyA RNA-seq with seedling shoots of NB and SR86 before and after 24-h salt treatment (Fig. 1a), with three biological repeats for each treatment (Additional file 1: Table S1). Before ribo-seq library construction, we examined polysome profiles of untreated and salt-treated samples. No significant differences were observed between the untreated and salt-treated samples or between the two cultivars (Additional file 2: Fig. S1). The quality of the ribo-seq libraries was evaluated by examining the reproducibility, size, distribution on genomic elements and 3-nt periodicity of the RPFs. Clustering analysis for these rice ribo-seq libraries revealed that the three biological repeats of NB or SR86 under normal and salt stress conditions were reproducible, though the distance between Repeat 1 and the other two repeats was larger than that between Repeat 2 and Repeat 3 (Fig. 1b and c). This moderate reproducibility was mainly attributed to the batch effects because the ribo-seq libraries of Repeat 1 and the other two repeats were prepared separately for both cultivars under normal and salt stress conditions. We then analyzed the size of RPFs in these samples and found that the RPFs ranged from 26 to 30 nt, with the majority of samples peaking at the previously reported RPF size of 28 nt (Additional file 2: Fig. S2A) [31, 39]. Although the peak size varied, these RPFs displayed a strong 3-nt periodicity with offset values of 11 or 12 nt to their corresponding P-sites (Fig. 1d; Additional file 2: Figs. S2B and S3). Consistent with previous results in *Arabidopsis* and tomato [31, 32], the rice RPFs were mainly derived from ORFs, as opposed to introns or untranslated regions (Additional file 2: Fig. S2C). These features indicate that high-quality ribo-seq libraries were successfully constructed for both rice cultivars. The reproducibility of polyA RNA-seq libraries was revealed for the three biological repeats of NB or SR86 under normal and salt stress conditions as well (Additional file 2: Fig. S4).

### Comparison of rice transcriptome and translatome under salt stress

**Fig. 2:**
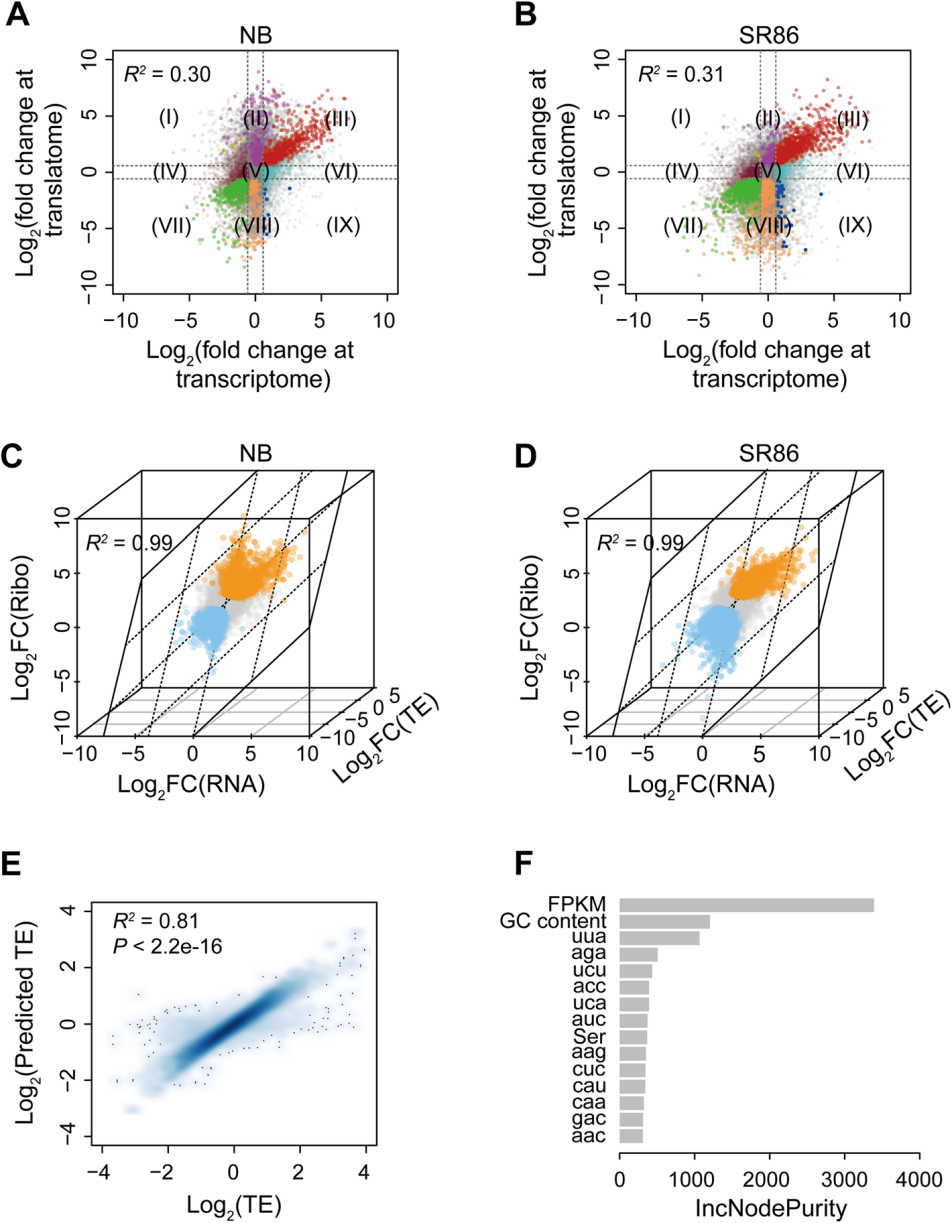
Comparison between transcriptome and translatome of ‘Nipponbare’ (NB) and ‘Sea Rice 86’ (SR86). **a** The correlation between the changes of gene expression at transcriptional and translational levels in NB under salt stress. **b** The correlation between the changes of gene expression at transcriptional and translational levels in SR86 under salt stress. Genes are categorized into nine groups based on their changes in the transcriptome and/or the translatome: I) genes that are transcriptionally down-regulated but translationally up-regulated, II) genes that are only translationally up-regulated, III) genes that are transcriptionally and translationally up-regulated, IV) genes that are only transcriptionally down-regulated, V) genes that are unchanged, VI) genes that are only transcriptionally up-regulated, VII) genes that are transcriptionally and translationally down-regulated, VIII) genes that are only translationally down-regulated, and IX) genes that are transcriptionally up-regulated but translationally down-regulated. **c** Translational alterations under salt stress are well explained by the combined changes of transcriptome and translation efficiency in NB. **d** Translational alterations under salt stress are well explained by the combined changes of transcriptome and translation efficiency in SR86. The translationally up- and down-regulated genes are colored in orange and cyan, respectively. **e** Correlation between the measured and predicted translation efficiency in rice. The predicted translation efficiency is derived from a random forest regression, which is trained by a dataset including codon usage frequency, amino acid usage frequency, coding sequence length, GC content and transcriptional level of each gene in rice. In (c)-(e), “TE” represents translation efficiency. **f** The top 15 most important sequence features that contribute to rice translation efficiency. The importance of these features is measured by the Mean Decreased Accuracy (IncNodePurity). “FPKM” is short for “fragments per kilobase of transcript per million fragments mapped”

To explore transcriptional and translational responses to salt stress, we compared fold changes that occurred between control and salt stress samples using both RNA-seq and ribo-seq data. The correlation coefficients of fold changes between transcriptome and translatome datasets were 0.30 for NB and 0.31 for SR86, both of which differed from the expected correlation coefficient (1.0) based on the assumption that the transcriptomic and translatomic changes under salt stress were completely concordant (Fig. 2a and b). We defined genes with fold change > = 1.5 and *P*-value <= 0.01 as being significantly differentially expressed at transcriptional and/or translational levels. With these criteria, all detected genes in NB and SR86 were categorized into nine groups: I) genes that were transcriptionally down-regulated but translationally up-regulated, II) genes that were only translationally up-regulated, III) genes that were transcriptionally and translationally up-regulated, IV) genes that were only transcriptionally down-regulated, V) genes that were unchanged, VI) genes that were only transcriptionally up-regulated, VII) genes that were transcriptionally and translationally down-regulated, VIII) genes that were only translationally down-regulated, and IX) genes that were transcriptionally up-regulated but translationally down-regulated (Fig. 2a and b; Additional file 3: Table S2; Additional file 4: Table S3). Group IV (2344 for NB and 3244 for SR86) and Group VI (2152 for NB and 2269 for SR86) contained the majority of significantly differentially expressed genes, followed by Group II (1415 for NB and 1880 for SR86), Group VIII (1355 for NB and 1860 for SR86), Group III (766 for NB and 1446 for SR86), Group VII (828 for NB and 1338 for SR86), Group IX (53 for NB and 106 for SR86) and Group I (16 for NB and 27 for SR86). These results revealed a significant amount of discordance in rice transcriptomic and translatomic changes in response to salt stress.

Changes in translation could be attributed to changes in transcript abundance and/or translation efficiency. To determine the extent of transcriptional abundance and translational efficiency changes, we first performed a regression analysis on transcript abundance, translation efficiency and RPF abundance, and found that approximately 99% of salt-induced global alterations in translation were explained by the combined contributions of alterations in transcript abundance and translation efficiency (Fig. 2c and d). Next, to understand the independent contribution of changes in transcript abundance and translation efficiency, we calculated their partial coefficient, a parameter that evaluates the contribution of a single variable without the effects from other variables. This analysis revealed that alterations in transcript abundance and translation efficiency contributed 57 and 65% to the final translation changes in NB, and 60 and 63% to the final translation changes in SR86 under salt stress, respectively (Table 1). In line with the strong contribution from translation efficiency, we also found a significant difference in translation efficiency between normal and salt stress conditions for both NB and SR86 (Wilcoxon test, *P*-value <= 0.01) (Table 2).

**Table 1:** Variables that contribute to translational changes in ‘Nipponbare’ (NB) and ‘Sea Rice 86’ (SR86) in response to salt stress

Cultivar	Contribution^a^	***R***^***2***b^
NB	Total	0.9996
	Common	−0.2189
	Transcription	0.5681
	Translation efficiency (TE)	0.6504
SR86	Total	0.9978
	Common	−0.2333
	Transcription	0.5989
	TE	0.6322

^a^ The combined contributions of transcription and TE alterations to changes in translation (Total) are partitioned into changes in transcription alone, TE alone, or both (common)

^b^ “*R*^*2*^” represents “Coefficient of determination”

**Table 2:** Comparison of translation efficiency (TE) across samples of ‘Nipponbare’ (NB) and ‘Sea Rice 86’ (SR86)

Sample^a^	Overall TE	***P***-value (Wilcoxon test)^b^
NB 0 h	1.139164	3.306e-05
NB 24 h	1.178219	
SR86 0 h	1.252071	< 2.20e-16
SR86 24 h	0.9922721	

^a^ “0 h” and “24 h” represent rice samples under normal and salt stress conditions, respectively

^b^*P*-value represents the statistical significance of the difference in TE between normal and salt stress conditions in NB and SR86

### Contributions of gene coding sequence features to translation efficiency

Due to the importance of translation efficiency in gene translational alterations, we further explored the influences of sequence features on translation efficiency in rice. Codon usage frequency, amino acid usage frequency, coding sequence length, GC content and transcript level (fragments per kilobase of transcript per million fragments mapped, FPKM) of each gene were used to build a training dataset, which was applied to a random forest model for translation efficiency prediction in rice. The predicted translation efficiency was able to fit the measured translation efficiency well (*R*^*2*^ = 0.81 and *P*-value < 2.2e-16) (Fig. 2e). The independent contribution of each variable was evaluated, and FPKM, GC content and UUA usage frequency were found as the top three most important contributors to alterations in translation efficiency (Fig. 2f). The association of specific sequence features with translation efficiency and protein abundance has previously been reported in *Arabidopsis* [40] and maize [23].

### Comparison of translational responses to salt stress between NB and SR86

**Fig. 3:**
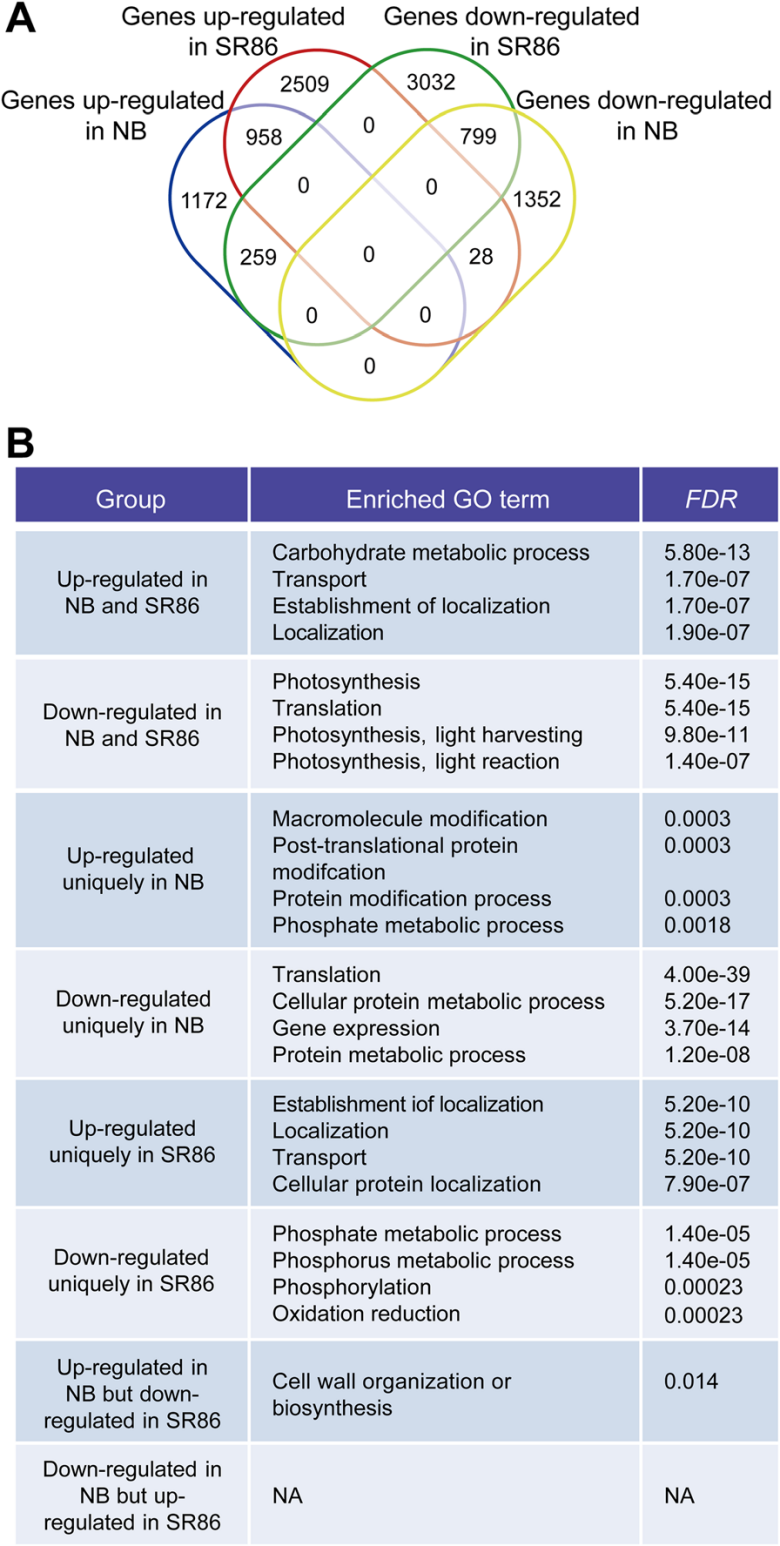
Comparison of translational responses of genes between ‘Nipponbare’ (NB) and ‘Sea Rice 86’ (SR86) under salt stress. **a** Venn diagram showing gene sets commonly or uniquely up- and/or down-regulated in NB and SR86 under salt stress. **b** Identification of overrepresented gene ontology (GO) terms in each gene set. The cutoff value for the overrepresented GO terms is *FDR* < = 0.05. The top four overrepresented GO terms are displayed for the gene sets of “up-regulated in NB and SR86”, “down-regulated in NB and SR86”, “up-regulated uniquely in NB”, “down-regulated uniquely in NB”, “up-regulated uniquely in SR86” and “down-regulated uniquely in SR86”. “NA” is short for “not available”

Since SR86 displays higher salt tolerance, it may have differences in its translational responses to salt stress compared to NB. To test this hypothesis, we compared the genes that were translationally up- or down-regulated after salt treatment in either NB or SR86 and found 2389 and 3495 genes that were significantly up-regulated (fold change > = 1.5 and *P*-value <= 0.01) in NB and SR86, respectively (Fig. 3a; Additional file 5: Table S4). Additionally, there were 2179 and 4090 genes that were significantly down-regulated (fold change > = 1.5 and *P*-value <= 0.01) in NB and SR86, respectively (Fig. 3a; Additional file 5: Table S4). Among these genes, 958 and 799 were up- and down-regulated in both cultivars in response to salt stress (Fig. 3a; Additional file 5: Table S4). More differentially translated genes (DTGs) were observed in SR86, suggesting a stronger translational response to salt stress for SR86 than NB.

We further interrogated the functions of the DTGs in response to salt stress in NB and SR86 and found that DTGs that occurred in both cultivars were associated with stress-related gene ontology (GO) terms (*FDR* < = 0.05) such as “transport” and “response to abiotic stimulus” (up-regulated), as well as “photosynthesis” and “generation of precursor metabolites and energy” (down-regulated) (Fig. 3b; Additional file 6: Table S5). In addition to the commonly up-regulated genes, genes associated with GO terms (*FDR* < = 0.05) such as “transport”, “oxidation reduction” and “cell redox homeostasis” were uniquely up-regulated in SR86 (Fig. 3b; Additional file 6: Table S5). In NB, significantly enriched GO terms (*FDR* < = 0.05) such as “translation” and “cellular protein metabolic process” were observed for the uniquely down-regulated gene set (Fig. 3b; Additional file 6: Table S5). In addition to the photosynthesis-related DTGs that occurred in both cultivars, NB also had several unique photosynthesis-related DTGs, implying that it likely undergoes more significant changes in photosynthesis under salt stress compared with SR86 (Additional file 6: Table S5). We also found that there were a series of genes that were up-regulated in NB but down-regulated in SR86 (259 genes) or down-regulated in NB but up-regulated in SR86 (28 genes) (Fig. 3a; Additional file 5: Table S4). Genes that were up-regulated in NB but down-regulated in SR86 were found to be enriched for the GO term “cell wall organization or biogenesis” (Fig. 3b; Additional file 6: Table S5). These large divergences in DTGs indicate that NB and SR86 might adopt different translational strategies to cope with salt stress.

### Ribosome occupancy along coding sequences in NB and SR86

**Fig. 4:**
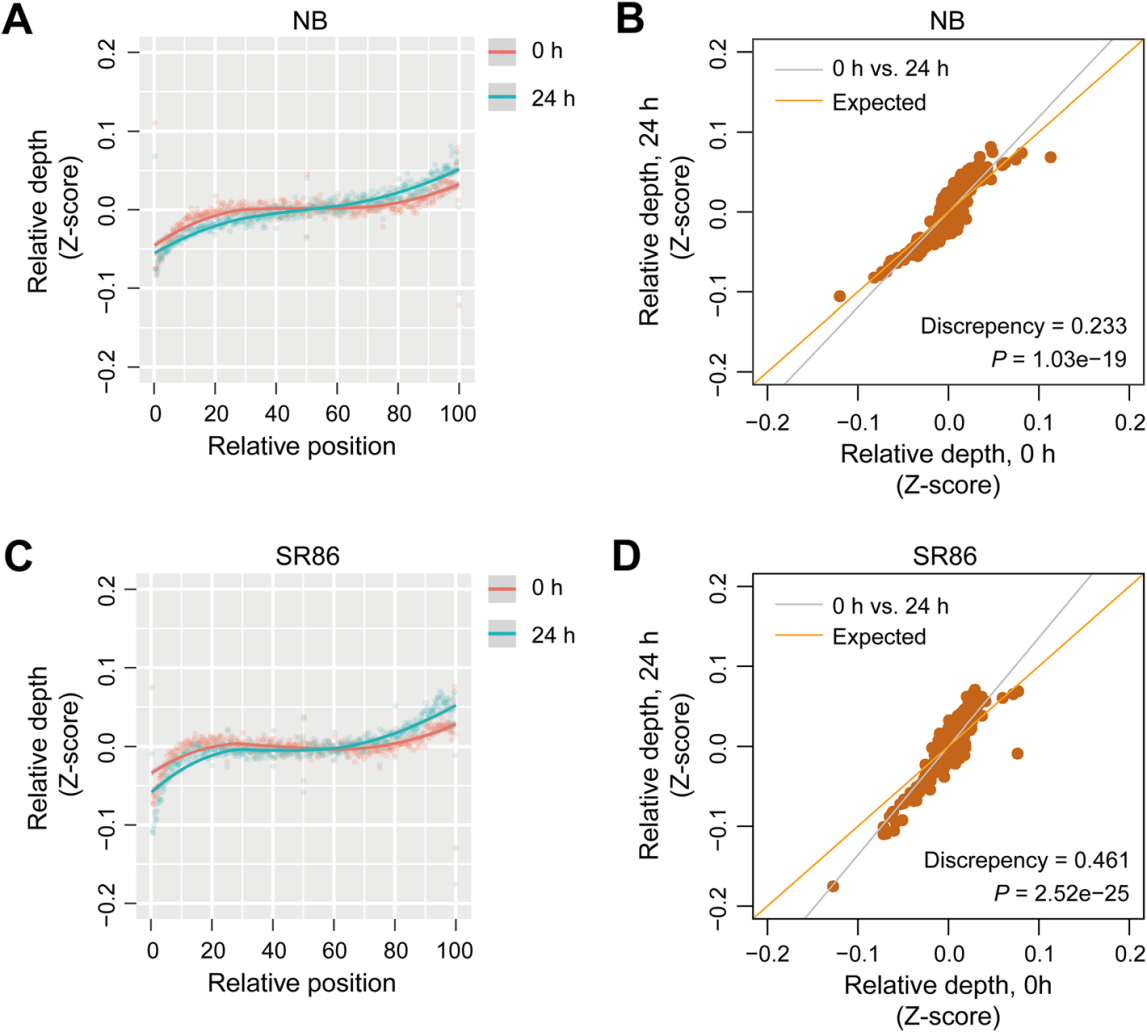
Comparison of ribosome-protected mRNA fragment (RPF) distribution along gene coding sequences in ‘Nipponbare’ (NB) and ‘Sea Rice 86’ (SR86). **a** The RPF distribution pattern derived from all genes in NB under normal (0 h, red line) and salt stress (24 h, blue line) conditions. **b** The coefficient of RPF depth of all detected genes between normal (0 h) and salt stress (24 h) conditions (grey line) is compared to the expectation of complete concordance between the two conditions (orange line) in NB. **c** The RPF distribution pattern derived from all genes in SR86 under normal (0 h, red line) and salt stress (24 h, blue line) conditions. **d** The coefficient of RPF depth of all detected genes between normal (0 h) and salt stress (24 h) conditions (grey line) is compared to the expectation of complete concordance between the two conditions (orange line) in SR86. In (a) and (c), higher Z scores indicate higher ribosome occupancy. The relative depth of RPFs is shown as the mean of three biological repeats

We mapped RPFs from NB and SR86 onto the rice coding sequences to explore ribosome occupancy globally. The varied translation levels among detected genes were normalized by converting RPF count to Z-score, and then the coding sequence of each gene was divided into 100 bins to indicate the relative positions along it. We found that RPFs were preferentially located at the 3′ regions and depleted at the 5′ regions of coding sequences in both NB and SR86 under normal and salt stress conditions (Fig. 4a and c). This pattern implies a faster ribosome movement at the 5′ regions of coding sequences and a gradually slower movement toward the 3′ ends of coding sequences, possibly due to the ribosome jam at stop codons (Fig. 4a and c).

This biased distribution was further strengthened in both cultivars by salt stress, particularly in SR86. As shown in Fig. 4a and c, at the 5′ halves of coding sequences, the normalized depth of RPFs was lower in both salt-treated rice cultivars (blue line) in comparison to untreated cultivars (red line). In addition, the depth of RPFs was higher at the 3′ halves of coding sequences in salt-treated rice compared to untreated rice. The 3′ shifted RPF distribution implies an accelerated movement of ribosomes in rice under salt stress. To evaluate whether the global ribosome occupancy observed in rice with and without salt stress was significantly different, we built a linear regression model with Z-scores of the two datasets in NB or SR86 and analyzed the differences between the observed regression coefficient and the expected one (1.0, indicating no difference). We found that there were significant differences in the ribosome occupancy between untreated and salt-treated rice, with a *P*-value of 1.03e-19 for NB and 2.52e-25 for SR86 (Fig. 4b and d).

Furthermore, comparison of RPF distribution on translationally up- and down-regulated genes was carried out in the two rice cultivars under normal and salt stress conditions, respectively. Significant shift toward the 3′ ends of coding sequences was observed for translationally up-regulated genes in SR86 (discrepancy = 0.539, *P-*value = 1.05e-19) but was not seen in NB (discrepancy = 0.00764, *P*-value = 0.871) (Additional file 2: Fig. S5A and B). The changes for translationally down-regulated genes in NB and SR86 were statistically significant, while the discrepancy of SR86 (0.196) was lower than that of NB (0.209) and the *P*-value of SR86 (1.04e-07) was much higher than that of NB (4.41e-13) (Additional file 2: Fig. S5C and D). These divergences in RPF distribution on the DTGs might partially explain the discrepancy in the global changes of ribosome occupancy between NB and SR86 under salt stress.

### Ribosome stalling in response to salt stress in NB and SR86

**Fig. 5:**
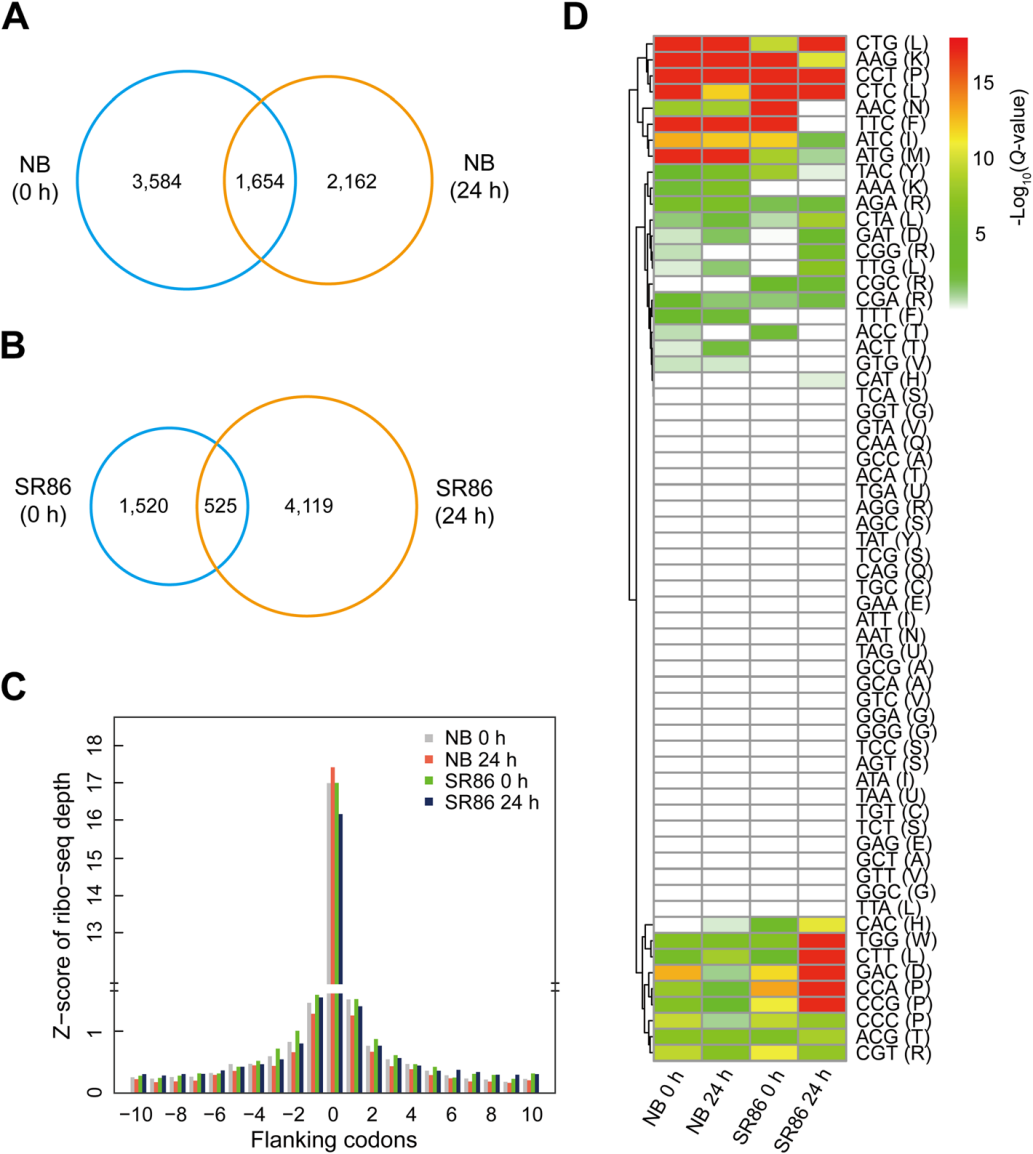
Analysis of ribosome stalling in ‘Nipponbare’ (NB) and ‘Sea Rice 86’ (SR86) in response to salt stress. **a** Venn diagram showing common or unique stalling sites in NB under normal (0 h) and salt stress (24 h) conditions. **b** Venn diagram showing common or unique stalling sites in SR86 under normal (0 h) and salt stress (24 h) conditions. **c** RPF depth at stalling sites and 20-codon flanking regions is shown in NB and SR86 under normal (0 h) and salt stress (24 h) conditions. The Z-score of ribo-seq depth is shown as the mean of three biological repeats. The number “0” in the x-axis indicates the ribosome stalling site. **d** The frequency of codons at ribosome stalling sites was compared with their corresponding frequency in gene coding sequences and a hypergeometric test was performed to identify codons with significantly higher occurrence (*Q*-value <= 0.01) at ribosome stalling sites. The *Q*-values of hypergeometric tests are shown in the heatmap

After normalizing RPF depth to Z-score for each gene, the features of ribosome stalling, including the number of stalling sites, stalling strength and codon usage, were monitored globally by detecting sites with Z-scores higher than 10 in three biological repeats. In total, we identified 5238 and 3816 ribosome stalling sites in NB, and 2045 and 4644 stalling sites in SR86 under normal and salt stress conditions, respectively (Fig. 5a and b). Among them, only a small portion (1654 for NB and 525 for SR86) were constantly occupied by stalled ribosomes under both normal and salt stress conditions, while most stalling sites were only detected either under normal or salt stress condition (Fig. 5a and b).

Thereafter, a global view of RPF occupancy at stalling sites together with 20-codon flanking regions was obtained by averaging the Z-scores of RPFs of all detected stalling sites and their flanking regions. Similar ribosome occupancy at stalling sites was observed between NB and SR86 under normal condition, while this occupancy was increased in NB but decreased in SR86 under salt stress (Table 3; Fig. 5c). With the increase in ribosome occupancy at the stalling sites, RPF depth was decreased at both upstream and downstream regions symmetrically in salt-treated NB (Fig. 5c). In contrast, ribosome occupancy at stalling sites was lowered in salt-treated SR86 with less influence on flanking regions (Table 3; Fig. 5c).

**Table 3:** Comparison of stalling strength at ribosome stalling sites across samples of ‘Nipponbare’ (NB) and ‘Sea Rice 86’ (SR86)

Comparison^a^	***P***-value (Kolmogorov-Smirnov test)
NB 0 h vs. NB 24 h	2.75e-11
SR86 0 h vs. SR86 24 h	< 2.20e-16
NB 0 h vs. SR86 0 h	0.9307
NB 24 h vs. SR86 24 h	< 2.20e-16

^a^ “0 h” and “24 h” represent rice samples under normal and salt stress conditions, respectively

We next sought to determine the frequency of codons at the sites of ribosome stalling and compared this with their genome-wide usage in NB and SR86. This analysis revealed that 21 codons (AAG, ATC, ATG, CCA, CCT, CGA, CGT, CTC, CTG, CTT, TGG, TTC, TTT, AGA, CCG, AAC, ACG, GAC, CCC, TAC and ACT) in NB and 23 codons (AAG, CCA, CCC, CCG, CCT, CGT, CTC, CTG, CTT, GAC, TGG, TTC, CGC, ATG, TAC, GAT, AAC, ATC, TTG, CTA, CAC, AGA and CGG) in SR86 occurred more frequently (*Q*-value <=0.01) at stalling sites as compared to their genome-wide usage under normal condition (Fig. 5d; Additional file 7: Table S6). Under salt stress, the number of codons significantly enriched (*Q*-value <=0.01) at ribosome stalling sites changed slightly to 23 (AAC, AAG, ACG, AGA, ATC, ATG, CCT, CGT, CTC, CTG, CTT, TAC, TGG, TTC, TTT, CCA, AAA, CTA, GAT, ACT, CCG, GAC and TTG) in NB and to 24 (AAG, CAC, CCA, CCC, CCG, CCT, CGT, CTC, CTG, CTT, GAC, GAT, TGG, TTG, CGG, CTA, AGA, ATG, CGC, CGA, CAT, TTC, AGG and ATC) in SR86 (Fig. 5d; Additional file 7: Table S6). Moreover, we observed that tRNAs recognized stalling codons at low to moderate levels in the two rice cultivars under normal and salt stress conditions (Additional file 2: Fig. S6A). However, the relationship between tRNA abundance and RPF depth at stalling codons was not linear (Additional file 2: Fig. S6B).

## Discussion

SR86, which was first discovered in the coastal area of South China in 1986, is a representative highly salt-tolerant rice germplasm that has been explored for breeding potential. This line has a sequenced genome and root transcriptome [16], but the molecular mechanisms underlying its salt tolerance have not been sufficiently explored. In the present study, we obtained new shoot transcriptomic and translatomic data for SR86. Additionally, we performed reference-guided assembly for SR86 and then compared the shoot transcriptomes of SR86 with NB to determine whether there were novel genes or transcripts in SR86. With the exception of 310 novel transcripts, all transcripts identified in SR86 had annotated counterparts in NB (Additional file 3: Table S2; Additional file 4: Table S3). Among these novel transcripts in SR86, no completely novel genes were identified. These results indicate that SR86 shares similar transcriptomic components with NB and thus their differences in salt tolerance are mainly attributed to differences in gene expression, which might be controlled at the transcriptional, post-transcriptional, translational or post-translational level. Moreover, this study revealed the global translational landscapes under normal and salt stress conditions in NB and provided a new translatomic resource for this intensively studied cultivar.

By comparing transcriptomic and translatomic responses to salt stress, we observed concordant changes in transcription and translation for many genes in both cultivars. However, a large proportion of genes displayed discordant alterations at the transcriptional and translational levels (Fig. 2a and b). For example, thousands of genes (Group IV and Group VI in Fig. 2a and b) in NB and SR86 were up- or down-regulated transcriptionally without significant alterations at the translational level. Genes with this transcription and translation pattern may undergo changes in translation later on if stress conditions persist [23, 41]. A large amount of genes (Group II and Group VIII in Fig. 2a and b) in NB and SR86 were up- or down-regulated at the translational level without changes in transcript abundance, indicating that these genes responded to stress conditions more rapidly at the translational level than at the transcriptional level [23]. Translational adjustment of this kind is considered a more rapid and direct strategy for environmental response [42]. A few genes (Group IX in Fig. 2a and b) were transcriptionally up-regulated but translationally down-regulated, possibly due to an increase in transcription coupled with the sequestration of translationally stalled mRNA into stress granules [43]. These transcripts could represent reserves that undergo translation when stress conditions are relieved or eliminated [44]. The largely discordant regulation between transcriptomes and translatomes in rice under salt stress was consistent with previous findings that gene expression was controlled in a relatively independent manner at transcriptional and translational levels in *Arabidopsis* under hypoxia and phosphate deficiency conditions [22, 24] and in maize under drought stress [23].

The two cultivars differed in the degrees of translational alterations in response to salt stress. More translationally up- and down-regulated genes were observed in SR86 than in NB under salt stress (Fig. 3a), suggesting better flexibility of translational regulation networks in SR86. Flexibility in gene regulation networks is considered an essential component of plant survival under unfavorable environmental conditions because it facilitates the reprogramming of physiological, metabolic and developmental processes [45, 46]. Furthermore, a large number of genes exhibited cultivar-specific translational alterations (Fig. 3a). For example, the expression of *OsRS1* (*LOC_Os01g13210*), which has previously been shown to be associated with salt tolerance [47], was translationally up-regulated in SR86, whereas no significant changes in its expression were detected in NB under salt stress (Additional file 5: Table S4). Similarly, the enhanced expression of *OsRS1* has been reported in seedling shoots of ‘Pokkali’, a salt-tolerant rice cultivar, in comparison to that in seedling shoots of the salt-sensitive cultivar ‘IR64’, when being challenged by salt stress [48], indicating that *RS1*-based salt tolerance might be a conserved acclimation mechanism. The identification of *RS1* gene in SR86 provides a new target for rice salt tolerance improvement. An additional set of antioxidant enzyme genes, such as *LOC_Os01g57730* encoding a peroxidase precursor and *LOC_Os05g25850* encoding a mitochondrial superoxide dismutase precursor, were observed to be significantly up-regulated in SR86 (Fig. 3b; Additional file 5: Table S4), suggesting a more active oxidation-reduction system in SR86 than in NB. Salt stress can impose both Na^+^ toxicity and osmotic stress, which result in abnormal accumulation of ROS [3]. Higher expression of antioxidant enzyme genes can lead to more ROS scavenging and protect cells from oxidative damage, resulting in increased plant survival under salt stress [49, 50]. *LOC_Os07g46460*, a gene encoding a ferredoxin-dependent glutamate synthase, was translationally up-regulated in SR86, while no significant alteration was observed for its translation in NB in response to salt stress (Additional file 5: Table S4). Higher expression of this gene has also been detected in the salt-tolerant rice ‘Pokkali’ with respect to the salt-sensitive rice ‘IR64’ [48]. The ferredoxin-dependent glutamate synthase is responsible for glutamate biosynthesis in plant leaves and plays a crucial role in photosynthesis by enabling re-assimilation of toxic photorespiration-derived ammonium ions [51–53]. In contrast, an additional set of photosynthetic genes such as *LOC_Os07g22498* and *LOC_Os10g21198*, which encode photosystem I iron-sulfur center protein and photosystem II reaction center protein K precursor respectively, were uniquely down-regulated at the translational level in NB under salt stress (Fig. 3b; Additional file 5: Table S4), suggesting a more sensitive photosynthetic system in NB than in SR86. Similarly, reduced expression levels of photosynthesis-related genes have been reported in the salt-sensitive rice cultivar ‘IR64’ as compared to the salt-tolerant rice cultivar ‘Pokkali’ [48]. These differences in translational adaptation might be important reasons for the differing salt tolerance between NB and SR86.

Ribo-seq data provide not only information on the translation efficiency of genes but also ribosome occupancy along the ORFs, thus shedding light on translational dynamics. To better understand the translational dynamics of salt-sensitive and -tolerant rice, we investigated ribosome occupancy in NB and SR86 under normal and salt stress conditions. A biased distribution of RPFs was observed on coding sequences, with a gradual increase of RPF depth from the 5′ to the 3′ ends of transcripts under normal condition in the two rice cultivars. Salt stress strengthened this biased distribution, particularly in SR86 (Fig. 4), indicating that ribosomes might be more dynamic in SR86. The more dynamic ribosomes may enable more rapid responses to salt stress at the translational level, and we found that more genes were translationally up- and down-regulated in SR86 than in NB under salt stress (Fig. 3a; Additional file 5: Table S4).

Translation initiation is typically thought to be the rate-limiting step for protein synthesis [42, 54–56]. However, increasing evidence points to the control of translation elongation by ribosome stalling as playing a critical role [57]. In the present study, we explored ribosome stalling in two rice cultivars under normal and salt stress conditions, and found that ribosome occupancy at flanking regions of stalling sites was influenced by ribosome stalling (Fig. 5c). In *E. coli*, a model termed “ribosome queuing” has been proposed to interpret the effects of ribosome stalling on ribosome occupancy at regions upstream and downstream of the stalling sites [58]. According to this model, ribosome stalling can result in the stacking of upstream ribosomes, whereas downstream ribosomes can still progress, resulting in decreased ribosome occupancy at downstream regions. Intriguingly, our results showed that upstream and downstream ribosomes at 20-codon flanking regions were distributed around the stalling sites in a symmetric pattern in both rice cultivars instead of ribosome queuing. Ribosome occupancy sharply diminished from the stalling sites to the first neighboring upstream and downstream codons and then deceased gradually to further upstream and downstream regions under both normal and salt stress conditions (Fig. 5c). Moreover, different alterations in the strength of ribosome stalling were observed in salt-sensitive and -tolerant cultivars in response to salt stress. Salt stress enhanced the strength of ribosome stalling in NB and further diminished upstream and downstream ribosomes, whereas it decreased ribosome occupancy at the stalling sites with little influence on ribosome distribution at flanking regions in SR86 (Table 3; Fig. 5c). Previous studies have demonstrated that ribosome drop-off, a translation abortion mechanism at the elongation phase, is closely associated with ribosome stalling and tends to occur more frequently on mRNAs without ribosome queuing in comparison to those with ribosome queuing [59, 60]. Thus, ribosome drop-off may partially explain the observed ribosome stalling with symmetrically diminishing ribosomes flanking the stalling sites. Ribosome stalling is generally accepted as having a negative impact on local translation elongation, ribosome recycling and protein synthesis [28, 61]. We therefore propose that these different ribosome stalling behaviors might be an important reason for the different translational dynamics between NB and SR86 under salt stress.

There are three possible explanations for the occurrence of opposite ribosome stalling variations in NB and SR86 under salt stress. Firstly, there might be distinct variations in specific aminoacyl tRNA amounts in both rice cultivars when being challenged by salt stress. In the present study, although most of the codons at the stalling sites corresponded to tRNAs with low abundance under normal condition, no apparent responses were detected for these tRNA species under salt stress (Additional file 2: Fig. S6A). No linear relationship was found between tRNA abundance and RPF depth at the stalling codons as well (Additional file 2: Fig. S6B). Therefore, variations in aminoacyl-tRNA amount do not fully explain the opposite stalling responses to salt treatment in NB and SR86. Ribosome stalling is also impacted by the biochemical nature of amino acids themselves. Previous studies have shown that, in comparison to other amino acids, proline is a poor A-site peptidyl acceptor and a poor P-site peptidyl donor due to the existence of an amino functional group and extraordinarily low reactivity with puromycin [62, 63], resulting in more frequent ribosome stalling [63, 64]. We found that ribosome stalling occurred at codons responsible for proline in the two rice cultivars under normal and salt stress conditions (Fig. 5d; Additional file 7: Table S6). However, some differences in the proline-induced ribosome stalling were discovered. Under normal condition, the four proline-related codons (CCA, CCG, CCT and CCC) displayed strong ribosome stalling in SR86, whereas only CCT and CCC displayed strong ribosome stalling in NB. Increased strength of ribosome stalling was further observed at the four proline-related codons in SR86 under salt stress, whereas stalling strength decreased at three proline-related codons (CCA, CCG and CCC) in salt-treated NB (Fig. 5d; Additional file 7: Table S6). Given the fact that the stalling sites globally displayed significantly increased strength in NB while they had reduced strength in SR86 under salt stress (Table 3; Fig. 5c), a small proportion of stalling sites, such as proline-related ones, cannot become a reasonable explanation for the observed variations in stalling strength in both rice cultivars that were challenged by salt stress. The third possible reason for ribosome stalling variations might be the distinct translational availability of amino acids in salt-stressed NB and SR86. Previous studies have shown that plants commonly accumulate amino acids under abiotic stresses, the majority of which are preferentially allocated to tolerance-related processes, such as osmotic adjustment, signal transduction and mitochondrial respiration instead of gene translation, to increase survival rate [65–69]. In comparison to stress sensitive rice, both the concentration and number of amino acids have been observed to be significantly enhanced in stress-tolerant rice under various stress conditions [70, 71]. We speculate that the greater accumulation of amino acids in stress-tolerant plants might satisfy the demand of amino acids for not only the tolerance-related processes but also gene translation, which is supported by our observations of more DTGs and greater RPF occupancy on transcripts in SR86 than in NB exposed to salt stress (Figs. 3a and Fig. 4). Therefore, the opposite variations in ribosome stalling strength are most likely attributed to the distinct amino acid profiles in salt-stressed NB and SR86.

**Fig. 6:**
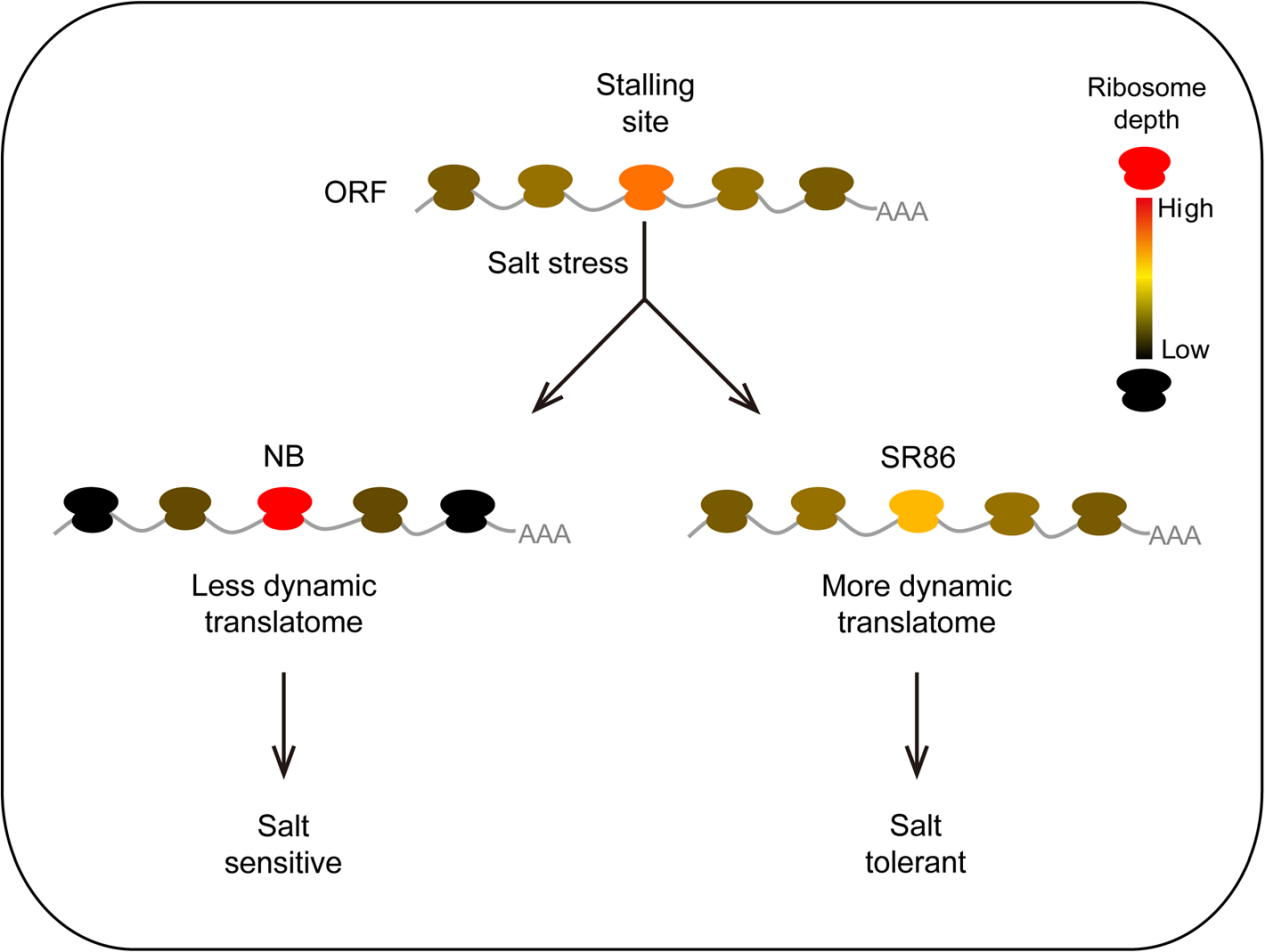
A proposed model for ribosome stalling-mediated translational adaption to salt stress in rice. In NB, a salt-sensitive rice cultivar, salt stress decreases the translational availability of amino acids and thus leads to increased ribosome stalling, which induces ribosome drop-off on flanking regions and decreases ribosome dynamics, leading to a reduction in translation. In salt-tolerant rice SR86, the global availability of amino acids for the translation process is enhanced and thus ribosome stalling is alleviated without significantly altering ribosome occupancy on flanking regions, implying more dynamic ribosomes. As a result, protein synthesis is regulated more rapidly and efficiently in SR86, thus allowing for better growth performance of SR86 under salt stress

Based on our observations, together with previously reported results, we generated a model to explain ribosome stalling-mediated translational adaptation to salt stress in rice (Fig. 6). In NB, a salt-sensitive cultivar, salt stress might decrease the translational availability of amino acids and thus enhance the strength of ribosome stalling, which further induces ribosome drop-off and decreases ribosome dynamics, resulting in reduced protein synthesis and translational adaption. In contrast, in SR86, a salt-tolerant cultivar, the global availability of amino acids for the translation process alleviates ribosome stalling, which results in more dynamic ribosomes. As a result, protein synthesis could be regulated more rapidly and efficiently, allowing for better growth performance of SR86 under salt stress.

## Conclusions

By performing ribosome profiling, we have provided new translatomic resources and revealed the translational landscapes for NB, a salt-sensitive and intensively studied cultivar, and SR86, a representative salt-tolerant cultivar. Our results show that translational reprogramming represents an important layer of salt stress responses in rice, and SR86 adopts a more flexible translationally adaptive strategy to cope with salt stress in comparison to NB because of its more dynamic ribosomes. Furthermore, we demonstrate that the differences in ribosome dynamics between SR86 and NB may be attributed to their differing strengths of ribosome stalling. Our study sheds new light on rice translational responses to salt stress and may provide potential targets at the translational level for engineering salt-tolerant cultivars in the future.

## Methods

### Plant materials and growth conditions

Rice (*O. sativa* L.) cultivars ‘Nipponbare’ (NB, salt-sensitive) and ‘Sea Rice 86’ (SR86, salt-tolerant) were used in this study. After germination, the seedlings were placed in hydroponic boxes containing Yoshida solution [72] in a growth chamber with the following settings: photoperiod of 12 h, air temperature of 28 °C for the light period and 25 °C for the dark period, and relative humidity of 60–70%. The hydroponic solution was refreshed at 3-day intervals, and rice seedlings of both cultivars were subjected to 150 mM NaCl treatment at the three-leaf stage. Seedling shoots were collected before and after 24-h salt treatment, and immediately frozen in liquid nitrogen for library construction. Three biological repeats were performed for each sample.

### Polysome isolation and profiling

Polysomes were isolated by the method described by Yang et al. [73]. Briefly, about 1 g of pulverized NB or SR86 shoots in liquid nitrogen was transferred to a 15-mL nuclease-free centrifuge tube with 5 mL ice-cold polysome extraction buffer (PEB) [100 mM Tris-HCl (pH 8.0), 40 mM KCl, 20 mM MgCl_2_, 2% (V/V) polyoxyethylene-10-tridecyl ether, 0.2% (W/V) deoxycholic acid, 1 mM DTT, 100 μg mL^− 1^ cycloheximide and 10 U mL^− 1^ DNaseI] and mixed well. The suspension was then centrifuged at 4 °C with a speed of 5000 g for 10 min, followed by another 20,000 g centrifugation at 4 °C for 10 min in a new 15-mL tube. The supernatant was subjected to polysome profile analysis and RPF isolation.

To perform profile analysis, the isolated polysome sample was loaded on a 15–60% (W/V) sucrose gradient in a polypropylene centrifuge tube (13 × 51 mm, BECKMAN, USA) and then centrifuged in a SW-55 rotor (BECKMAN, USA) at 4 °C with a speed of 170,000 g for 1.5 h. Fractionation, absorbance assay and data acquisition of the resulting gradient were carried out using a gradient fractionator system (BRANDEL, USA) with a UA-6 absorbance detector (TELEDYNE ISCO, USA).

### RPF preparation

Prior to library construction, the RNA concentration of polysome extracts was first adjusted to 400 ng μL^− 1^. For isolation of RPFs, the 200 μL aliquots were subjected to nuclease (ARTseq/TruSeq Mammalian Ribo Profile Kit, ILLUMINA, USA) digestion (20 U per 40 μg of RNAs) at 25 °C for 1.4 h in a dry bath (THERMO, USA) with an agitation speed of 600 rpm. After terminating the digestion with 15 μL of SUPERase-in (AM2696, THERMO, USA), the resulting product was immediately placed on an Illustra MicroSpin S-400 HR column (GE HEALTHCARE, USA) that was equilibrated with 3 mL of PEB to collect the monosomes. RPFs were then extracted with the SDS method, and further purified by Zymo RNA clean and concentrator kits R1017 and R1015 (ZYMO RESEARCH, USA), respectively. The filtrate was subjected to rRNA removal with the Ribo-Zero rRNA Removal Kit for Plant Leaf (MRZPL1224, ILLUMINA, USA). Finally, RPFs around 28–30 nt were recovered by PAGE purification as described previously [73].

### Library construction

Ribo-seq libraries for NB and SR86 were constructed with the recovered RPFs, and then subjected to PCR enrichment with TruSeq Ribo Profile Forward and Index primers, according to previously described method [73]. The PCR products were purified with AMPure XP beads (BECKMAN, USA), followed by 8% (W/V) native PAGE. The bands ranging from 140 to 160 bp were excised from the native PAGE and libraries were recovered from the excised gel pieces by overnight elution [73].

To construct RNA-seq libraries, 50 μL of the adjusted shoot polysome extracts were treated with SDS solution, followed by purification with a Zymo RNA clean and concentrator kit R1017 (ZYMO RESEARCH, USA) for total RNA extraction. Then, 5 μg of RNA samples were subjected to rRNA removal with the Ribo-Zero rRNA Removal Kit for Plant Leaf (MRZPL1224, ILLUMINA, USA). Using the rRNA-depleted samples, RNA-seq libraries were constructed with the ARTseq/TruSeq Ribo Profile Kit (Illumina), and enriched by PCR amplification with TruSeq Ribo Profile Forward and Index primers. Purification and recovery of the PCR products were done by following the previously described protocol for ribo-seq library construction.

Both RNA-seq and ribo-seq libraries were sequenced on an ILLUMINA HiSeq2500 platform with the single-end 50-bp sequencing strategy (BERRY GENOMICS, BEIJING, CHINA).

### Analysis of differentially expressed genes

The raw reads of RNA-seq or ribo-seq were subjected to adaptor trimming and then mapped to the rice MSU7.0 reference genome (http://rice.plantbiology.msu.edu/) using Cutadapt v1.15 [74] and Bowtie1 [75], respectively. After discarding reads that were aligned to rRNAs or tRNAs, the number of reads aligned to each gene was counted for the identification of differentially transcribed/translated genes (fold changes > = 1.5 and *P*-values <= 0.01) using the R package DESeq2 [76]. GO term analysis for differentially translated genes was conducted using agriGO (http://bioinfo.cau.edu.cn/agriGO/) with default parameters, and the terms with *FDR* < = 0.05 were considered as significantly enriched ones.

### Analysis of translation efficiency

The normalization for both the RNA-seq and ribo-seq reads aligned to each gene was carried out by using a middle-ratio algorithm implemented in DESeq2 [76]. Translation efficiency, which was defined as the ratio of normalized read count of ribo-seq to that of RNA-seq for each ORF, was then calculated. Comparison of the overall translation efficiency between samples was performed with Wilcoxon test.

To assess the independent contribution of RNA abundance and translation efficiency to salt-induced translation changes in NB and SR86, the coefficient of determination (*R*^2^) was partitioned into three contributions, *ρ*^2^(*FC-Ribo*, *FC-RNA* or *FC-TE*), *ρ*^2^(*FC-Ribo*, *FC-RNA* | *FC-TE*) and *ρ*^2^(*FC-Ribo*, *FC-TE* | *FC-RNA*). *FC-Ribo*, *FC-RNA* and *FC-TE* represent the fold changes of translation, transcription and translation efficiency of ORFs, respectively, under salt stress observed in NB and SR86, and *ρ*^*2*^(*FC-Ribo*, *FC-RNA* | *FC-TE*) is the independent contribution of *FC-RNA* in which the contribution from *FC-TE* is excluded. The semi-partial correlation was then calculated according to a formula described previously [77].

### Metagene analysis

After trimming adaptors, the periodicity of RPFs was first evaluated using the R package “Multitaper” [78]. It was expected that representative RPFs displayed 3-nt periodicity on the transcripts, similar to a wave with 1/3 Hz periodicity. Therefore, RPFs with strong periodicity and correct frequency were considered to have a significant (*P*-value <= 0.01) periodicity at 1/3 Hz. Metagene analysis was then carried out using RiboCode [79] with default parameters.

### RPF distribution and ribosome stalling

After mapping RPFs to coding sequences, the translation P-sites were allocated according to their offsets in metagene analysis. To make RPF locations comparable on different genes, normalization was carried out for the positions of RPFs by the following formula:

$$ \mathrm{Relative}\ \mathrm{Position}=\frac{100\times \mathrm{RPF}\ \mathrm{position}}{\mathrm{Length}\ \mathrm{of}\ \mathrm{CDS}} $$

To exclude the bias of gene expression, the RPF depth at P-sites of each coding sequence was also normalized by converting them to Z-scores, according to the following formula:

$$ \mathrm{Z}-\mathrm{score}=\frac{\mathrm{RPF}\ \mathrm{counts}\ \mathrm{at}\ \mathrm{each}\ \mathrm{base}-\mathrm{Mean}\ \mathrm{of}\ \mathrm{RPF}\ \mathrm{counts}}{\mathrm{Standard}\ \mathrm{deviation}\ \mathrm{of}\ \mathrm{RPF}\ \mathrm{counts}} $$

Sites with Z-scores greater than 10 in three biological repeats were considered as ribosome stalling sites and kept for further analysis. To compare RPF distribution patterns, linear regressions were done for the Z-scores between samples from each pair, and the differences in coefficients were statistically analyzed between different regressions. In detail, the Z-scores from NB or SR86 under normal and salt stress conditions were converted into a vector of X and Y, respectively, from which a linear regression was made using R, and the coefficient of the model was then compared to 1.0. The null hypothesis that the RPF distribution patterns from the two samples were the same was rejected if the coefficient was significantly different from 1.0 (*P*-value <= 0.01).

The codons at ribosome stalling sites were extracted, and their appearance frequency was computed and compared with their genome-wide usage. A hypergeometric test was performed, and codons with significantly higher appearance at ribosome stalling sites (*Q*-value <= 0.01) were identified by comparing to their appearance at the coding regions of the genome.

### Quantification of tRNAs

RNA-seq reads were mapped to the MSU7.0 reference genome (http://rice.plantbiology.msu.edu/) and those mapped only to tRNA loci were retained. The percentage of reads mapped to tRNAs was calculated and used to represent the total abundance of tRNAs. To accurately quantify the abundance of each tRNA, we first counted the reads unique to each tRNA, and reads with multiple possible mapping sites were then assigned to each tRNA according to the ratio of uniquely mapped reads between them. Finally, the percentage of reads from each tRNA was used as a proxy of the abundance.

## Supplementary Information

**Additional file 1: Table S1.** Raw data of rice RNA-seq and ribo-seq libraries.

**Additional file 2: Fig. S1** Ribosome profiles along 15–60% (W/V) sucrose gradients in ‘Nipponbare’ (NB) and ‘Sea Rice 86’ (SR86). **(A-B)** Profiles of ribosomes from NB under normal condition (0 h, A) or after 24-h salt stress (24 h, B). **(C-D)** Profiles of ribosomes from SR86 under normal condition (0 h, C) or after 24-h salt stress (24 h, D). Ribosome profiles are obtained by recording absorbance at 254 nm during sucrose gradient fractionation (from the top to the bottom of gradient). **Fig. S2** Size distribution, periodicity and coverage on genomic elements of ribosome-protected mRNA fragments (RPFs) in ribo-seq libraries of ‘Nipponbare’ (NB) and ‘Sea Rice 86’ (SR86). **(A)** Size (in nucleotide, nt) distribution of RPFs in ribo-seq libraries of NB and SR86 under normal (0 h) and salt stress (24 h) conditions. **(B)** Periodicity (in Hz) analysis of RPFs in ribo-seq libraries of NB and SR86 under normal (0 h) and salt stress (24 h) conditions by the F-score test implemented in “Multitaper”, an R package. The horizontal dashed line indicates the cutoff for significant periodicity (*P*-value = 0.001) and the vertical dashed line shows the position of 1/3, the expected frequency (3-nt periodicity) of RPFs. **(C)** The percentage distribution of RPFs on exon, intron, 5′ UTR and 3′ UTR in the ribo-seq libraries of NB and SR86 under normal (0 h) and salt stress (24 h) conditions. “rep 1”, “rep 2” and “rep 3” represent the three biological repeats. **Fig. S3** Metagene analysis of ribosome-protected mRNA fragments (RPFs) in ribo-seq libraries of ‘Nipponbare’ (NB) and ‘Sea Rice 86’ (SR86). **(A-D)** Metagene analysis of RPFs in ribo-seq libraries of NB under normal (0 h, repeat 2 for A and repeat 3 for B) and salt stress (24 h, repeat 2 for C and repeat 3 for D) conditions. **(E-H)** Metagene analysis of RPFs in ribo-seq libraries of SR86 under normal (0 h, repeat 2 for E and repeat 3 for F) and salt stress (24 h, repeat 2 for G and repeat 3 for H) conditions. Lines at positions of frame 0 (the main frame based on the annotated start codon), 1 and 2 are colored in purple, cyan and orange, respectively. **Fig. S4** Clustering analysis of transcriptomic datasets from ‘Nipponbare’ (NB) and ‘Sea Rice 86’ (SR86). **(A)** Clustering analysis of transcriptomic datasets from NB under normal (0 h) and salt stress (24 h) conditions. **(B)** Clustering analysis of transcriptomic datasets from SR86 under normal (0 h) and salt stress (24 h) conditions. “rep 1”, “rep 2” and “rep 3” represent the three biological repeats. The color schemes indicate Euclidean distances between samples measured by DESeq2-normalized read counts. **Fig. S5** Comparison of ribosome-protected mRNA fragment (RPF) distribution along gene coding sequences in ‘Nipponbare’ (NB) and ‘Sea Rice 86’ (SR86). **(A-B)** The coefficient of RPF depth of translationally up-regulated genes between normal (0 h) and salt stress (24 h) conditions (grey line) is compared to the expectation of complete concordance between the two conditions (orange line) in NB (A) and SR86 (B). **(C-D)** The coefficient of RPF depth of translationally down-regulated genes between normal (0 h) and salt stress (24 h) conditions (grey line) is compared to the expectation of complete concordance between the two conditions (orange line) in NB (C) and SR86 (D). The relative depth of RPFs displays as the mean of three biological repeats. **Fig. S6** tRNA abundance in RNA-seq libraries of ‘Nipponbare’ (NB) and ‘Sea Rice 86’ (SR86). **(A)** tRNA abundance proxied by the percentage of reads mapped to each tRNA loci in NB and SR86 under normal (0 h) and salt stress (24 h) conditions. **(B)** The correlation between the strength of ribosome stalling and tRNA abundance.

**Additional file 3: Table S2.** Differentially transcribed/translated genes under salt stress in seedling shoots of ‘Nipponbare’ (NB).

**Additional file 4: Table S3.** Differentially transcribed/translated genes under salt stress in seedling shoots of ‘Sea Rice 86’ (SR86).

**Additional file 5: Table S4.** Differentially translated genes under salt stress in seedling shoots of ‘Nipponbare’ (NB) or ‘Sea Rice 86’ (SR86).

**Additional file 6: Table S5.** Complete lists of gene ontology (GO) terms for genes translationally up- and down-regulated under salt stress in ‘Nipponbare’ (NB) or ‘Sea Rice 86’ (SR86).

**Additional file 7: Table S6.** Comparison of codon appearance at ribosome stalling sites with their corresponding frequency in gene coding sequences in ‘Nipponbare’ (NB) and ‘Sea Rice 86’ (SR86).

## Data Availability

The data reported in this paper have been deposited in the NCBI Sequence Read Archive (SRA) database (https://www.ncbi.nlm.nih.gov/subs/sra) under accession no. PRJNA523300.

## Abbreviations

DTG: Differentially translated gene
FPKM: Fragments per kilobase of transcript per million fragments mapped
GO: Gene ontology
NB: Nipponbare
ORF: Open reading frame
QTL: Quantitative trait locus
PEB: Polysome extraction buffer
ROS: Reactive oxygen species
RPF: Ribosome-protected mRNA fragment
SOS: Salt overly sensitive
SR86: Sea rice 86
