# Supplementary figures and images for: Comparative ribosome profiling reveals distinct translational landscapes of salt-sensitive and -tolerant rice
Source: BMC Genomics. 2021 Aug 12;22:612. doi: 10.1186/s12864-021-07922-6 (PMC8359061; doi:10.1186/s12864-021-07922-6)

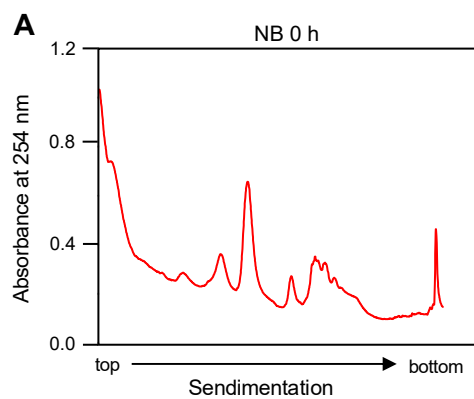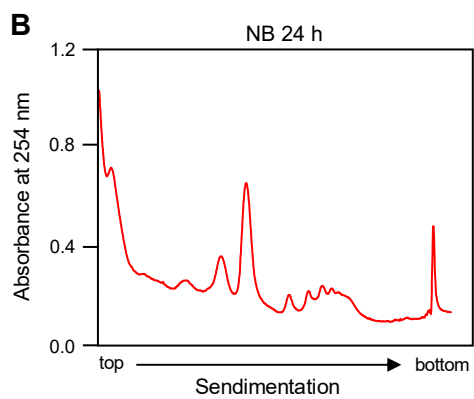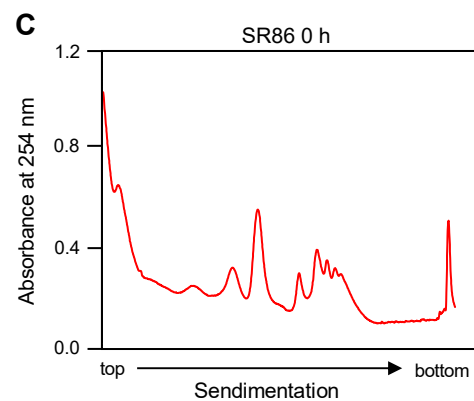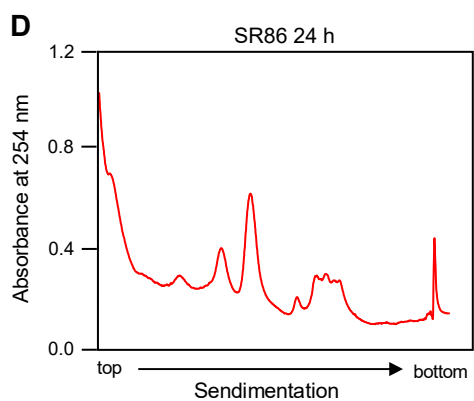

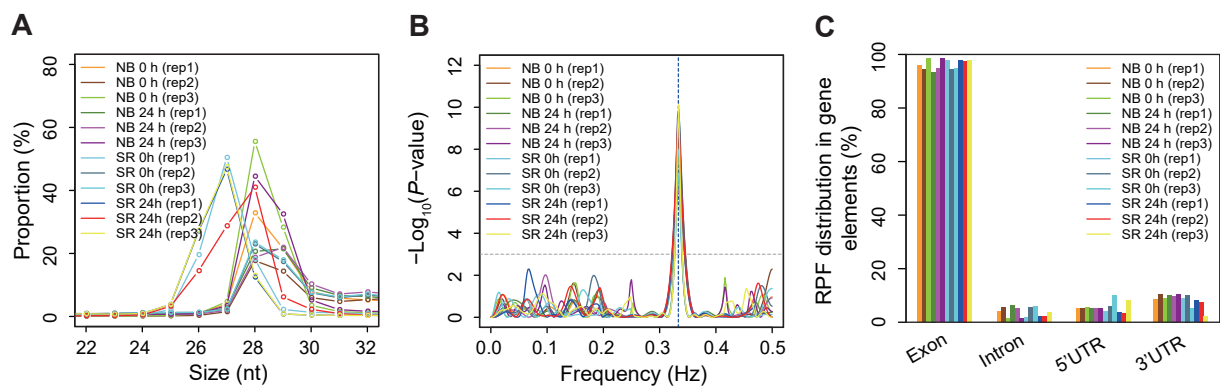

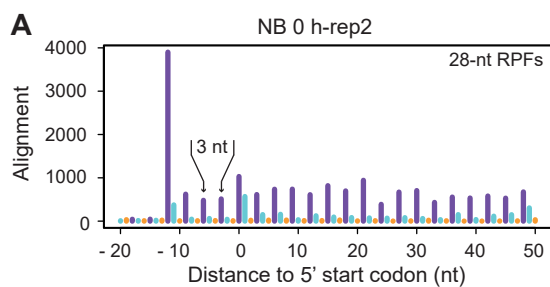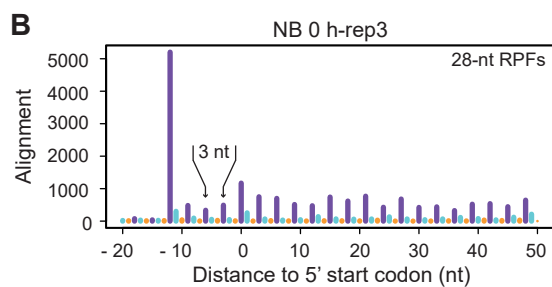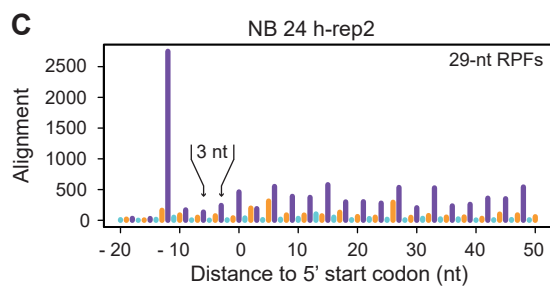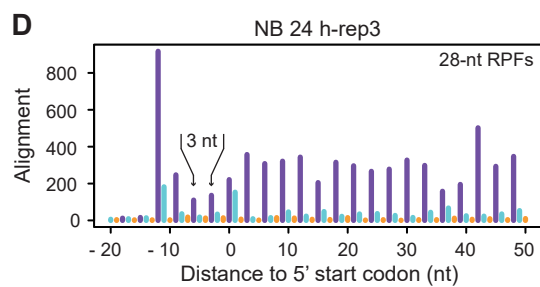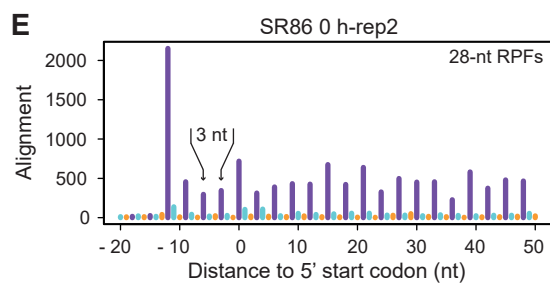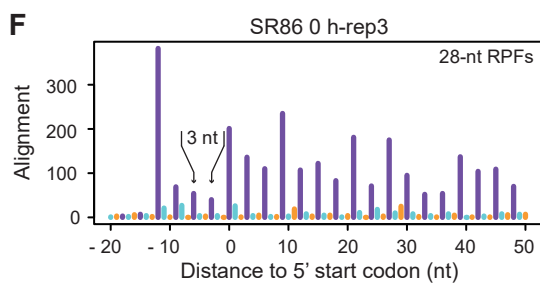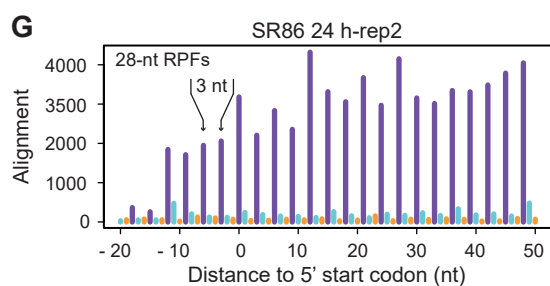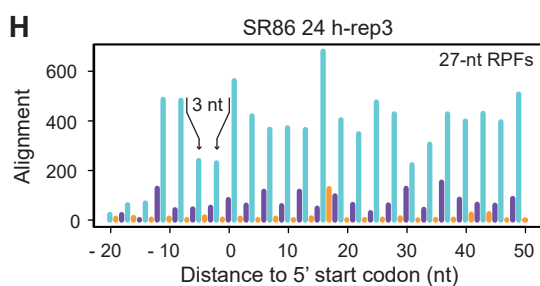

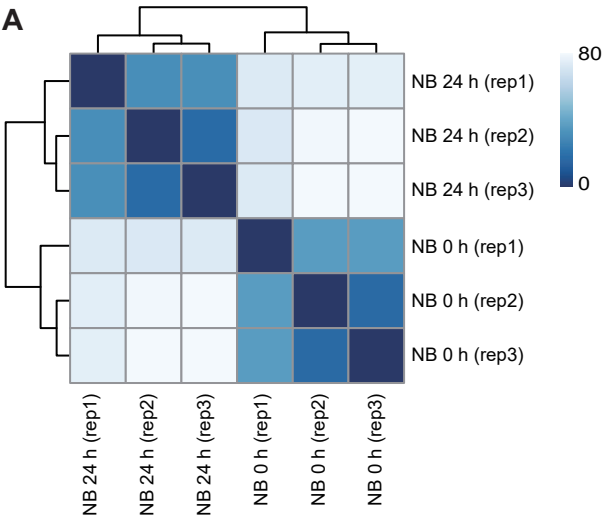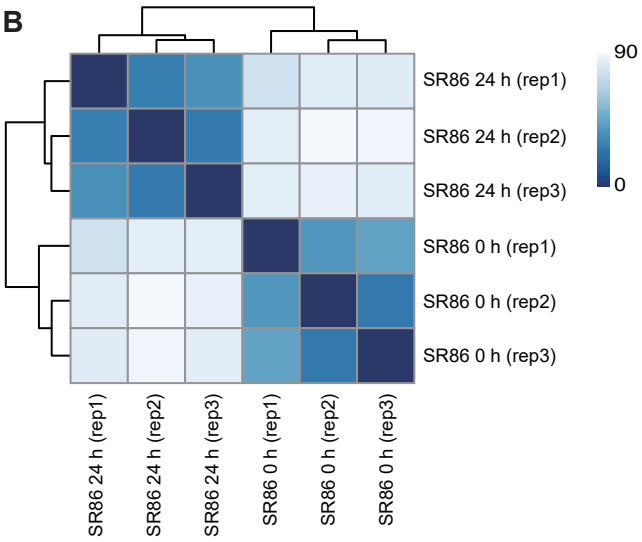

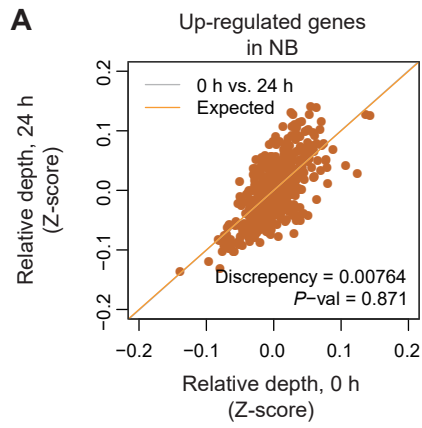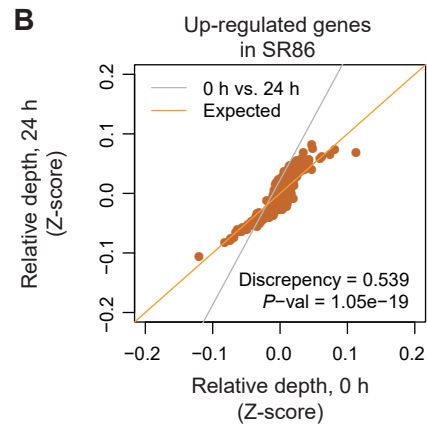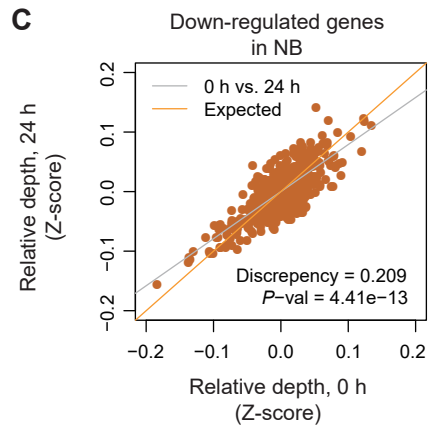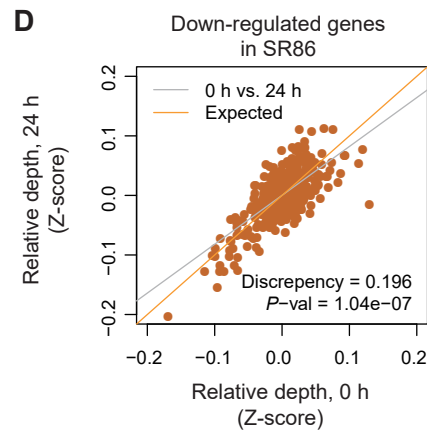

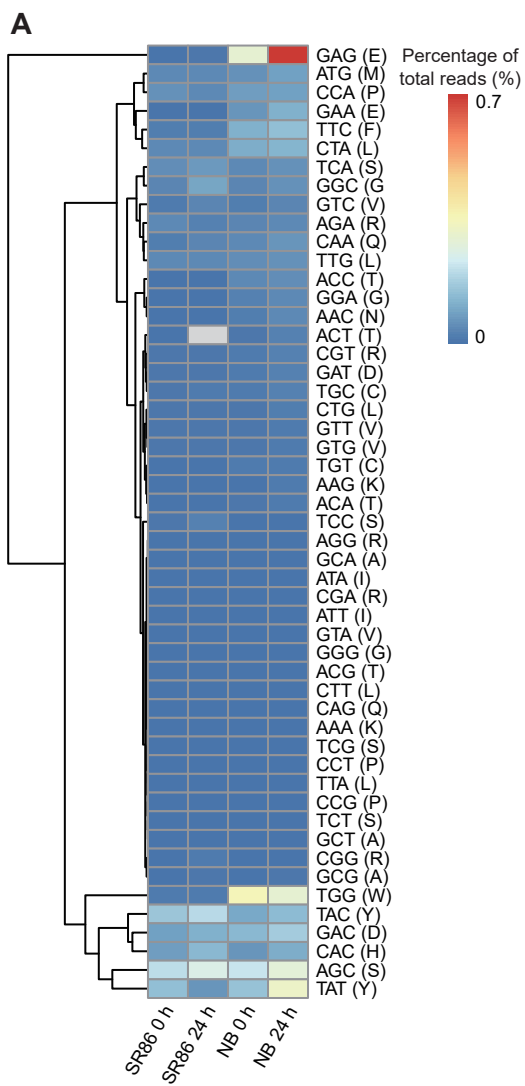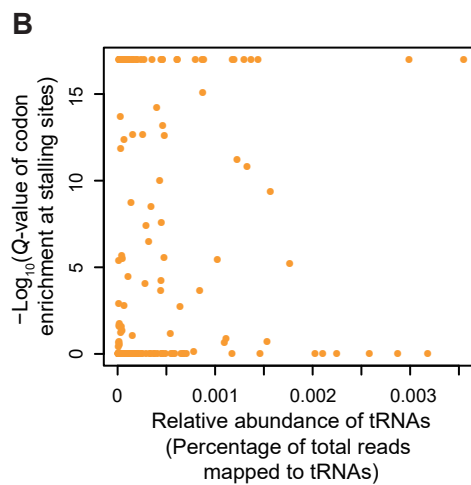

Supplement: Supplementary file 2 — Additional file 2: Fig. S1 Ribosome profiles along 15–60% (W/V) sucrose gradients in ‘Nipponbare’ (NB) and ‘Sea Rice 86’ (SR86). (A-B) Profiles of ribosomes from NB under normal condition (0 h, A) or after 24-h salt stress (24 h, B). (C-D) Profiles of ribosomes from SR86 under normal condition (0 h, C) or after 24-h salt stress (24 h, D). Ribosome profiles are obtained by recording absorbance at 254 nm during sucrose gradient fractionation (from the top to the bottom of gradient). Fig. S2 Size distribution, periodicity and coverage on genomic elements of ribosome-protected mRNA fragments (RPFs) in ribo-seq libraries of ‘Nipponbare’ (NB) and ‘Sea Rice 86’ (SR86). (A) Size (in nucleotide, nt) distribution of RPFs in ribo-seq libraries of NB and SR86 under normal (0 h) and salt stress (24 h) conditions. (B) Periodicity (in Hz) analysis of RPFs in ribo-seq libraries of NB and SR86 under normal (0 h) and salt stress (24 h) conditions by the F-score test implemented in “Multitaper”, an R package. The horizontal dashed line indicates the cutoff for significant periodicity (P-value = 0.001) and the vertical dashed line shows the position of 1/3, the expected frequency (3-nt periodicity) of RPFs. (C) The percentage distribution of RPFs on exon, intron, 5′ UTR and 3′ UTR in the ribo-seq libraries of NB and SR86 under normal (0 h) and salt stress (24 h) conditions. “rep 1”, “rep 2” and “rep 3” represent the three biological repeats. Fig. S3 Metagene analysis of ribosome-protected mRNA fragments (RPFs) in ribo-seq libraries of ‘Nipponbare’ (NB) and ‘Sea Rice 86’ (SR86). (A-D) Metagene analysis of RPFs in ribo-seq libraries of NB under normal (0 h, repeat 2 for A and repeat 3 for B) and salt stress (24 h, repeat 2 for C and repeat 3 for D) conditions. (E-H) Metagene analysis of RPFs in ribo-seq libraries of SR86 under normal (0 h, repeat 2 for E and repeat 3 for F) and salt stress (24 h, repeat 2 for G and repeat 3 for H) conditions. Lines at positions of frame 0 (the main frame [file 12864_2021_7922_MOESM2_ESM.pdf]
